# Influence of Vitamin C on Antioxidant Capacity of In Vitro Perfused Porcine Kidneys

**DOI:** 10.3390/nu11081774

**Published:** 2019-08-01

**Authors:** Christian Bleilevens, Benedict M. Doorschodt, Tamara Fechter, Tim Grzanna, Alexander Theißen, Elisa A. Liehn, Thomas Breuer, René H. Tolba, Rolf Rossaint, Christian Stoppe, Peter Boor, Aileen Hill, Gregor Fabry

**Affiliations:** 1 Department of Anesthesiology, Medical Faculty RWTH Aachen University Hospital, D-52074 Aachen, Germany; 2 Institute for Laboratory Animal Science & Experimental Surgery, Medical Faculty RWTH Aachen University Hospital, D-52074 Aachen, Germany; 3 Department of Thoracic and Cardiovascular Surgery, Medical Faculty RWTH Aachen University Hospital, D-52074 Aachen, Germany; 4 Department of Intensive Care Medicine and Intermediate Care, Medical Faculty RWTH Aachen University Hospital, D-52074 Aachen, Germany; 5 Institute for Molecular Cardiovascular Research (IMCAR), Medical Faculty RWTH Aachen University Hospital, D-52074 Aachen, Germany; 6 Human Genetic Laboratory, University of Medicine and Pharmacy, RO-200000 Craiova, Romania; 7 Institute of Pathology & Division of Nephrology, Medical Faculty RWTH Aachen University Hospital, D-52074 Aachen, Germany

**Keywords:** acute kidney injury, kidney transplantation, oxidative stress, antioxidant, vitamins, ascorbic acid, porcine kidney perfusion model in vitro techniques, animal models, organ dysfunction, reperfusion injury, primary graft dysfunction

## Abstract

Systemic and localized ischemia and reperfusion injury remain clinically relevant issues after organ transplantation and contribute to organ dysfunctions, among which acute kidney injury is one of the most common. An in vitro test-circuit for normothermic perfusion of porcine kidneys after warm ischemia was used to investigate the antioxidant properties of vitamin C during reperfusion. Vitamin C is known to enhance microcirculation, reduce endothelial permeability, prevent apoptosis, and reduce inflammatory reactions. Based on current evidence about the pleiotropic effects of vitamin C, we hypothesize that the antioxidant properties of vitamin C might provide organ-protection and improve the kidney graft function in this model of ischemia and reperfusion. Methods: 10 porcine kidneys from 5 Landrace pigs were perfused in vitro for 6 h. For each experiment, both kidneys of one animal were perfused simultaneously with a 1:1 mixture of autologous blood and modified Ringer’s solution at 38 °C and 75 mmHg continuous perfusion pressure. One kidney was treated with a 500 mg bolus injection of vitamin C into the perfusate, followed by continuous infusion of 60 mg/h vitamin C. In the control test circuit, an equal volume of Ringer’s solution was administered as a placebo. Perfusate samples were withdrawn at distinct points in time during 6 h of perfusion for blood gas analyses as well as measurement of serum chemistry, oxidative stress and antioxidant capacity. Hemodynamic parameters and urine excretion were monitored continuously. Histological samples were analyzed to detect tubular- and glomerular-injury. Results: vitamin C administration to the perfusate significantly reduced oxidative stress (49.8 ± 16.2 vs. 118.6 ± 23.1 mV; *p* = 0.002) after 6 h perfusion, and increased the antioxidant capacity, leading to red blood cell protection and increased hemoglobin concentrations (5.1 ± 0.2 vs. 3.9 ± 0.6 g/dL; *p* = 0.02) in contrast to placebo treatment. Kidney function was not different between the groups (creatinine clearance vit C: 2.5 ± 2.1 vs. placebo: 0.5 ± 0.2 mL/min/100 g; *p* = 0.9). Hypernatremia (187.8 ± 4.7 vs. 176.4 ± 5.7 mmol/L; *p* = 0.03), and a lower, but not significant decreased fractional sodium excretion (7.9 ± 2 vs. 27.7 ± 15.3%; *p* = 0.2) were observed in the vitamin C group. Histological analysis did not show differences in tubular- and glomerular injury between the groups. Conclusion: Vitamin C treatment increased the antioxidant capacity of in vitro perfused kidney grafts, reduced oxidative stress, preserved red blood cells as oxygen carrier in the perfusate, but did not improve clinically relevant parameters like kidney function or attenuate kidney damage. Nevertheless, due to its antioxidative properties vitamin C might be a beneficial supplement to clinical kidney graft perfusion protocols.

## 1. Introduction

Improving the quality of kidney grafts is currently one of the main challenges, as the increasing demand for donor organs clearly exceeds the availability. In 2016, there were 33,291 adult patients removed from the kidney waiting lists worldwide; with over one fourth deceased due to aggravated medical condition during the waiting time for transplantation, which reflects the donor organ shortage [1]. Numerous organs, which have been rejected due to their extended criteria donors, or donation after cardiac death status in the past might have been rescued or will be rescued in future if improved preservation strategies become available. These organs suffer from extended warm ischemia time and the aggravated reperfusion injury.

The major challenge is to overcome the factors inducing kidney graft dysfunction, which is mainly related to Ischemia and reperfusion (I/R)-injury with oxidative stress and inflammation [2], [3,4,5]. During reperfusion after a period of ischemia, the amount of reactive oxygen species can increase dramatically thereby exceeding the natural antioxidant defenses, causing damage to macromolecules and thus significantly injuring cell structures and function on a local, as well as the systemic level [6]. This results in a systemic inflammatory response syndrome, manifesting as pyrexia, tachycardia, leukocytosis, hypotension, edema, and organ failure [2]. Moreover, hypoperfusion, over- or underhydration, usage of nephrotoxic drugs, endotoxemia and cholesterol emboli can add to tubular injury, edema and kidney injury.

During the last decades, several strategies for preservation of kidney grafts prior to transplantation have been developed. As a golden standard, cold storage is applied, and different research concepts, such as the combination with hypothermic machine perfusion preceding, following or in between cold storage have been focused [7]. However, during recent years normothermic machine perfusion (NMP) of porcine [8] or human kidneys [9] emerged as the most promising strategy, enabling the successful transplantation of human kidney grafts that were previously declined [10]. Yong et al. postulated that NMP might have the potential to increase the donor pool by improving the outcome after organ transplantation of organs from extended criteria donors or those from donations after cardiac death [11].

Our working group recently published a study showing significant benefits of six hours direct NMP without previous cold flushing on porcine kidney function and damage (FABRY et al.). Kidneys preserved by NMP immediately after explanation were compared to cold flushed grafts according to clinical protocols before NMP was initiated. Regardless of the observed beneficial effects (i.e., improved creatinine clearance, reduced osmotic nephropathy, decreased urine protein concentration), we stated that the perfusate, which was in accordance to commonly used recipes [12,13], needs further improvement to extend the benefits of NMP without cold flush- or storage beyond six hours of perfusion.

Vitamin C is an essential, pleiotropic, and water-soluble micronutrient required for more than 60 enzymatic reactions, among which are the synthesis of norepinephrine collagen and carnitine [14,15]. Vitamin C is involved in iron absorption, peptide amination, tyrosine and steroid metabolism and cytochrome P450-driven hydroxylation of aromatic drugs and carcinogens [16]. Vitamin C enhances cell differentiation from somatic cells to induced pluripotent stem cells [17], which might play an important role for regenerating processes in patients undergoing major surgery (e.g., kidney transplantation), as well as in isolated organs. Vitamin C is known to restore vascular responsiveness to vasoconstrictors [18], ameliorates microcirculatory blood flow, preserves endothelial barriers [19], prevents apoptosis and augments the bacterial defense [20]. Based on its redox-potential and powerful antioxidant capacity, vitamin C has been described as the most important antioxidant, especially in I/R injury [21]. Vitamin C demonstrated organoprotective effects in the nervous, cardiovascular, respiratory, gastrointestinal, coagulation and immune systems in preclinical as well as in clinical studies [16,22].

Therefore, we aimed to investigate the antioxidant capacity of vitamin C and its pleiotropic effects as an essential micronutrient for organ protection in an in vitro I/R-porcine kidney NMP model.

## 2. Methods

### 2.1. Animals

The experimental protocol was approved by the Institutional Animal Care and Use Committee of the Rheinisch-Westfälische Technische Hochschule (RWTH) Aachen University Hospital and performed in accordance with German legislation governing animal studies following the ‘Guide for the Care and Use of Laboratory Animals’ (National Institute of Health publication, 8th edition, 2011) and the Directive 2010/63/EU on the protection of animals used for scientific purposes (Official Journal of the European Union, 2010).

Five female German Landrace pigs with 64.4 ± 0.8 kg body weight (BW, mean ± SEM) were housed in fully air-conditioned rooms with 22 °C room temperature, and a relative humidity of 50%. After arrival, the pigs were allowed to acclimatize to their surroundings for a minimum of seven days and fasted for 12 h before surgery with free access to water. As premedication the animals received intramuscular injections of 8 mg/kg BW azaperone (Stresnil, Janssen-Cilag GmbH, Neuss, Germany), 15 mg/kg BW ketamine (Ceva GmbH, Duesseldorf, Germany) and 10 mg atropine (1 mL/1% atropine sulfate, Dr. Franz Köhler Chemie GmbH, Bensheim, Germany). The femoral vein of the anaesthetized animal was cannulated, and 600 mL of venous blood were withdrawn into two heparinized blood bags (5000 IU/bag, B. Braun Melsungen AG, Melsungen, Germany), before the animals were euthanized by an intravenous administration of 1 mL/kg BW pentobarbital (Narcoren, Merial GmbH, Hallbergmoss, Germany). Immediately after cardiac arrest, a midline laparotomy was performed, and both kidneys were explanted simultaneously to achieve an equal warm ischemic time. The warm ischemic time was defined as the duration from cardiac arrest until explanation. In compliance with the 3R principle (Replacement, Reduction and Refinement of animal experiments) [23], the kidneys for this study were obtained from animals which were initially used by another in-house working group and furthermore, the other organs of the animals were also used for different in vitro research purposes in different in-house institutes.

### 2.2. Test Circuits

Two identical in-vitro test-circuits for normothermic machine perfusion of porcine kidneys were used to investigate the effects of vitamin C in porcine kidneys. The perfusate was collected from the renal vein into a hard shell-reservoir (Capiox CR10NX, Terumo Deutschland GmbH, Eschborn, Germany), and circulated by a centrifugal pump through an oxygenator (Deltastream DP2, HILITE 800^®^; both MEDOS Medizintechnik AG, Stolberg, Germany) into the renal artery. A continuous pressure of 75 mmHg was maintained by a computer-controlled custom-built pump controller. The temperature was kept at 38 °C by a water bath thermostat. Perfusate flow and pressures were monitored continuously, using an ultrasonic flow probe (SonoTT, em-tec GmbH, Finning, Germany) and pressure transducers (DATEX AS/3, GE Healthcare; Solingen, Germany).

The test circuit was primed using 300 mL of autologous blood and 300 mL of modified Ringer’s solution. After connection of the kidney graft, the first 200 mL drainage from the renal vein were discarded, leaving a full circuit volume of 500 mL.

### 2.3. Kidney Perfusion and Vitamin C Administration

The kidney blood vessels and the ureter were cannulated immediately after explanation (renal artery catheter: retrograde cardioplegia catheter, 14 French, Edwards Life Sciences; Unterschleißheim, Germany/renal vein catheter: ¼” tube connector, ¼” tubing, free life medical GmbH, Aachen, Germany/ureter catheter: 14 French catheter; Convatec Germany GmbH, Munich, Germany). The kidneys were then connected to the test circuit and perfused at 75 mmHg mean arterial pressure for 6 h. Both kidneys of one animal were perfused simultaneously in two identical test circuits. One circuit received vitamin C for intravenous use (WOERWAG Pharma GmbH & Co. KG, Boeblingen, Germany) diluted in Ringer’s solution, while in the control circuit an equal amount of Ringer’s solution was administered; all other parameters, interventions and handling procedures were identical in both circuits. Kidneys were allocated to experimental groups (vitamin C or control) and test-circuits in a randomized manner, to prevent a bias. The vitamin C was stored at 7 °C and administered to the circuit with ultraviolet (UV)-protected infusion lines and syringes. The vitamin C circuit was primed with an initial bolus injection of 500 mg vitamin C into the perfusate immediately before connecting the kidneys, followed by continuous infusion of 60 mg/h. This dosage was chosen in line with the dose-finding study by Fowler et al., which was observed it to be most effective. As high-dose vitamin C administration—especially for longer times—may also have negative effects on kidneys, for example occurrence of kidney stones [24], our research group abstained from higher vitamin C dosages, such as 66 mg/kg/h [25] or 125 mg/kg [26], which have previously described by other authors in other clinical settings.

### 2.4. Sampling and Measurements

Blood samples were drawn at distinct points in time during 6 h of perfusion (0, 30, 60, 120, 240, 300 and 360 min) for blood gas analyses including hemoglobin (HB) and electrolytes (ABL800; Radiometer GmbH, Willich, Germany) and subsequent analysis. Markers of inflammation were assessed using appropriate ELISA kits (interleukin 6 (IL6), interleukin 10 (IL10), tumor necrosis factor-α (TNF-α); all from R&D Systems, Wiesbaden, Germany). The measurement of serum chemistry (serum and urine creatinine) was performed in the inhouse central laboratory. Hemolysis was detected using a colorimetric assay (hemoglobin FS (flüssig, stabil/liquid, stable) reagent; Diasys Inc, Holzheim, Germany). 25 µl of plasma was mixed with 85 µl of the kit-reagent in a 96 well plate format, and the absorption was detected on a microplate reader (iMark; BioRad Laboratories GmbH, Munich, Germany) at 540 (A1) and 680 nm (A2) wavelength. The concentration of free HB was calculated according to the formula (A1-A2) × 733 in mg/dL. Oxidative stress was assessed using the oxidation-reduction potential (ORP) and Antioxidant Capacity (AC), measured with the RedoxSYS Diagnostic SystemTM (Aytu BioScience, Inc., Englewood, CO, USA). A low ORP being a sum of all oxidants and reductants in the blood indicates low oxidative stress, while a high AC is a measure of good antioxidant defense. Hemodynamic parameters and urine excretion were monitored continuously. Renal tissue samples were processed and Periodic acid-Schiff (PAS) staining was performed as described previously [27], to analyze tubular- and glomerular-injury, using a scoring system (0 = no; 1 = mild; 2 = moderate; 3 = severe).

### 2.5. Statistical Analysis

Statistical analysis was performed using GraphPad Prism 8 software package (GraphPad Software Inc, La Jolla, CA, USA). A two-way analysis of variance (ANOVA) and multiple Comparison were used followed by Bonferroni post-test correction for all measurements during perfusion, after performing a Shapiro-Wilk normality test. The effects of time were calculated by multivariate analysis for repeated measurements. For the comparsion of histological damage scores between the groups one sample t-test was applied. Data are presented as mean ± SEM and a *p* value < 0.05 was considered statistically significant.

## 3. Results

### 3.1. Perfusion Parameters

**Figure 1:**
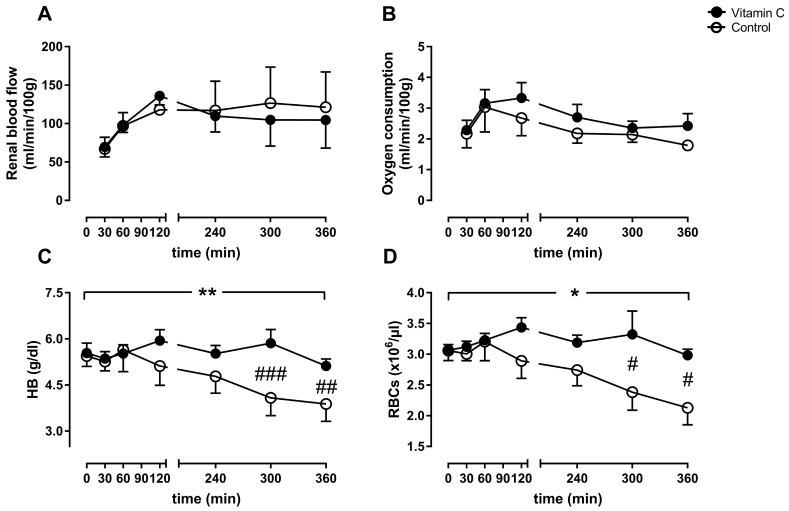
Perfusion parameters displayed as renal blood flow (**A**), oxygen consumption (**B**), arterial hemoglobin (HB, **C**), and the red blood cell count (RBCs, **D**). */** *p* < 0.05/0.01 effect of time for the control group; ### *p* < 0.001 vs. Control, ## *p* < 0.01 vs. Control, # *p* < 0.05 vs. Control.

Renal blood flow (Figure 1A) and oxygen consumption (Figure 1B) remained stable on comparable levels in both groups. In parallel to the significant decrease of HB over time in the control group (Figure 1C), we detected a significant decrease of the red blood cell count in the control group (Figure 1D). We could not detect stronger hemolysis in the control group. The free hemoglobin value after 6 h was comparable between the groups (vitamin C: 21.7 ± 9.3 vs. control: 12.1 ± 5.4 mg/dL; *p* = 0.37) (data not shown).

### 3.2. Kidney Function

**Figure 2:**
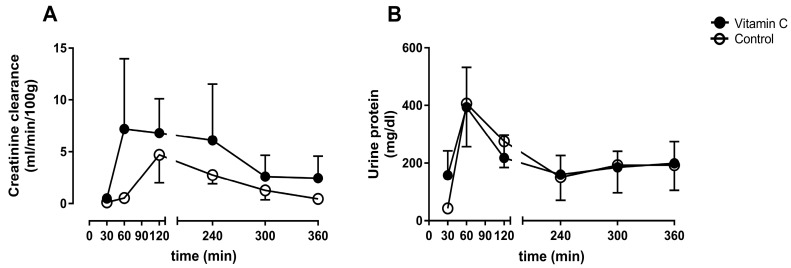
Creatinine clearance rate (**A**), and concentration of urine protein (**B**).

Kidney function was determined by creatinine clearance. The amount of urine protein over time represents a marker for kidney damage. Creatinine clearance was not improved in the vitamin C group in comparison to the control group at any time (Figure 2A). The concentration of urine protein remained on comparable levels between the groups during the whole experiment (Figure 2B).

### 3.3. Blood Gas Analyses

**Figure 3:**
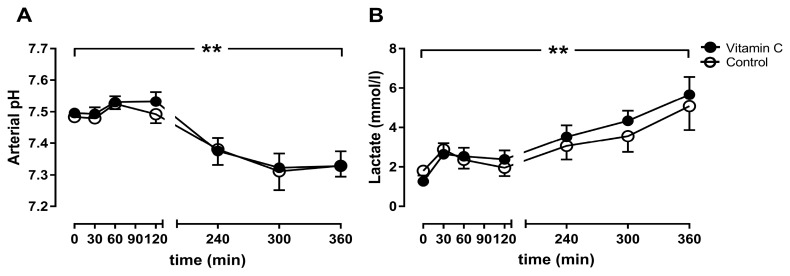
The arterial pH value (**A**) and the lactate Concentration (**B**). ** *p* < 0.01 effect of time for both groups.

The arterial pH decreased significantly in both groups over time from 7.49 ± 0.00 to 7.33 ± 0.02 after 6 h (vitamin C), and from 7.48 ± 0.01 to 7.33 ± 0.02 (control) (Figure 3A), without significant differences between the groups. In contrast, the lactate concentration increased significantly, from 1.26 ± 0.14 to 5.66 ± 0.4 mmol/L (vitamin C) and from 1.8 ± 0.23 to 5.08 ± 0.54 mmol (control), without any differences between the groups (Figure 3B).

**Figure 4:**
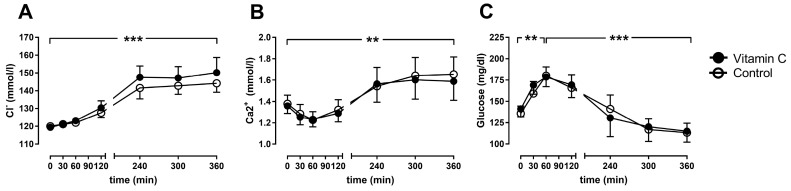
The arterial chloride- (**A**), calcium- (**B**) and glucose-concentrations (**C**). ** *p* < 0.01; *** *p* < 0.001 effect of time for both groups.

The arterial chloride concentration increased over time in both groups and trends to be higher in the vitamin C group after 6 h (150.2 ± 3.8 mmol/L) in comparison to the control group (144.2 ± 2.3 mmol/L; Figure 4A). Arterial calcium concentration increased over time, without differences between the groups (Figure 4B). The arterial glucose concentration increased during the first hour of perfusion in both groups, before it fell back to almost baseline levels in both groups (Figure 4C).

**Figure 5:**
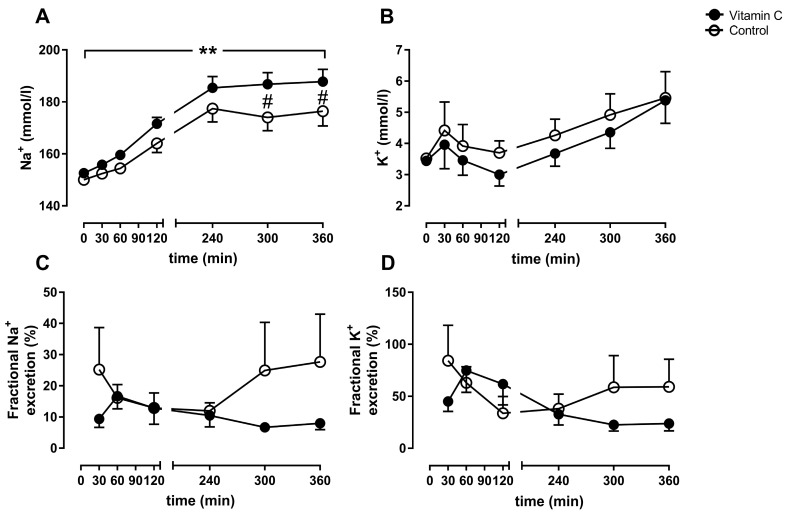
Arterial sodium- (**A**) and potassium-concentration (**B**). Fractional sodium- (**C**) and potassium-excretion (**D**). ** *p* < 0.01 effect of time for both groups; # *p* < 0.05 vs. Control.

The arterial sodium concentrations increased over time in both groups and reached significance between the groups at 5 h of perfusion (vitamin C: 186.8 ± 1.98 mmol/L; Control: 174 ± 2.3 mmol/L) and 6 h (vitamin C: 187.8 ± 2.12 mmol/L; Control: 176.4 ± 2.54 mmol/L) (Figure 5A). The arterial potassium concentrations increased over time without reaching significance within or between the groups (Figure 5B). The fractional sodium- and potassium-excretion was higher at 6 h of perfusion in the control group (sodium: 27.8 ± 6.6%; potassium: 59.1 ± 11.9%;) in comparison to the vitamin C group (sodium:7.9 ± 0.91%; potassium: 23.9 ± 3.2%) without reaching significance between the groups (sodium: *p* = 0.381, potassium: *p* = 0.341; Figure 5C/D).

### 3.4. Inflammation

**Figure 6:**
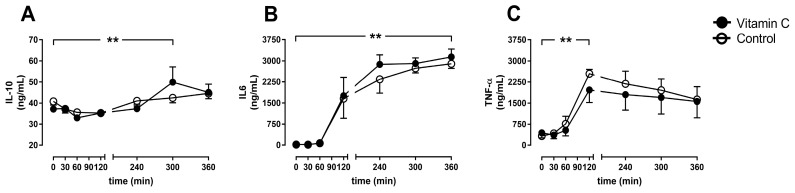
Interleukin 10 (IL10; **A**), IL6 (**B**), and Tumor necrosis factor-alpha (TNF-α; **C**). ** *p* < 0.01 effect of time for both groups.

The anti-inflammatory parameter IL10 increased in the vitamin C group until five hours of perfusion but falls back to comparable levels between the groups after 6 h without any differences between the groups (Figure 6A). The pro-inflammatory marker IL6 increased during the entire course of the experiments in both groups (Figure 6B), whereas the initiator of inflammatory response TNF-α peaked after two hours in both groups and decreased to lower levels until the end of the experiments (Figure 6C).

### 3.5. Oxidative Stress

**Figure 7:**
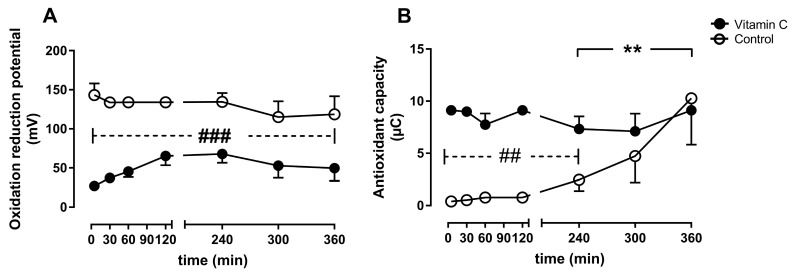
Oxidative stress was assessed as oxidation reduction potential (**A**), and antioxidant capacity (**B**). ** *p* < 0.01 effect of time for the control group; ## *p* < 0.01 vs. control, ### *p* < 0.001 vs. control.

The oxidation-reduction potential was significantly lower in the vitamin C group compared to the control group during the entire perfusion period (Figure 7A). The antioxidant capacity was higher in the vitamin C group at the beginning of perfusion and remained stable until the end, whereas the antioxidant capacity increased significantly from 4–6 h in the control group and reached comparable levels to the vitamin C group at the end of the experiment (Figure 7B).

### 3.6. Histological Analysis

The microscopic examination of PAS stained kidney cross sections after six hours perfusion revealed diffuse, acute tubular injury with tubular dilatation, anisometric vacuolization of the cytoplasm of tubular cells, and single cell necrosis in both groups, consistent with ischemia-reperfusion type of injury. No glomerular or vascular pathology was observed. The mean tubular injury score was similar in both groups, i.e., 2.0 ± 0.3 in the vitamin C group, compared to 1.8 ± 0.4 in the control group (*p* = 0.707).

## 4. Discussion

In this study, porcine kidneys were perfused in an extracorporeal pressure-controlled normothermic perfusion system, and vitamin C administration to the perfusate was compared to placebo treatment in two identical perfusion circuits. We could demonstrate a strong effect of vitamin C on oxidative stress and antioxidant capacity. However, vitamin C did not improve the creatinine clearance, renal blood flow, or oxygen consumption. The proinflammatory cytokines IL6 and TNF-α increased in both groups, whereas the anti-inflammatory cytokine IL10 was slightly increased in the vitamin C group. Interestingly, we found significantly higher hemoglobin concentrations at the end of the experiments in the vitamin C group, as the result of significantly higher red blood cells counts. The electrolyte concentrations, potassium, chloride and calcium did not differ between groups, but a significant hypernatremia was observed in the vitamin C group, resulting from decreased fractional sodium excretion. Histological examination showed acute tubular injury and osmotic vacuolization in both groups as typical signs for in vitro perfused kidneys without significant differences between groups.

The vitamin C group had a significantly lower oxidation reduction potential throughout the whole experiment, indicating lower oxidative stress, as well as higher antioxidant capacity during the early phase of reperfusion, indicating better defense against oxidative stress. These results confirm the well-described antioxidant capacities of vitamin C, however to our knowledge, it is the first time that these effects are described in an in vitro organ perfusion setting for kidney grafts. As the greatest ischemia- and reperfusion injury is expected shortly after reperfusion, it is not surprising that the antioxidant capacity recovers in both groups during the next hours. The recovery of the antioxidant capacity in the control group could possibly be explained via mobilization of intracellular reserves of antioxidant molecules into the bloodstream as reaction to the consumption of these molecules. However, the transport of antioxidants between cells and extracellular remains yet to be elucidated [26]. Whether the significant reduction of oxidative stress early after reperfusion will lead to improved kidney function reflected by creatine clearance and reduced kidney damage reflected in histologically observed damage remains unclear in our experiments, which might be due to the short duration of six hours.

The recovery of the antioxidant capacity in the control group could possibly be explained via mobilization of intracellular reserves of antioxidant molecules into the bloodstream as reaction to the consumption of these molecules. However, the transport of antioxidants between cells and extracellular remains yet to be elucidated [28].

The pronounced hypernatremia we detected in the vitamin C group, together with lowered sodium excretion might be explained by increased vitamin C storage, which is initiated via renal sodium-dependent vitamin C transporters (SVCT1, SVCT2). Vitamin C and sodium are co-transported into the cells in the proximal renal tubes, leading to reduced sodium excretion and higher arterial sodium content [29,30]. Thus, the treatment with a high vitamin C bolus dosage directly from the onset of organ perfusion, or systemic administration of vitamin C to the organ donor seem to be beneficial strategies to prepare the kidney for the detrimental effects of reperfusion after ischemia, by enhancing the intracellular vitamin C reserve.

The rapid increase in blood flow and oxygen consumption during the first hour of the perfusion initiates a strong I/R injury and inflammation cascade by inflow of immune cells into the warm-ischemia damaged tissue. Therefore, it would be of particular relevance in the initial phase of reperfusion to achieve a sufficient vitamin C supplementation in order to increase antioxidant defenses, by saturating the perfusate prior to reperfusion, as we applied in this study. A trend towards increased urine production was observed in the vitamin C group (data not shown), which might be clinically relevant when considering that delayed graft function is a predictor for impaired transplantation outcomes [31]. Although the vitamin C treatment, especially as a bolus at the beginning of perfusion, seems to improve kidney function during the later phase of the experiments, it did not confine the inflammatory response. The increase of TNF-α as proinflammatory marker during the initial phase was significant in comparison to baseline levels in both groups. Even though there was a more prominent peak of TNF-α in the control group, the increase of IL6 in the follow-up phase of inflammatory cytokine release was not stronger compared to the vitamin C group.

The most prominent effect of vitamin C we observed, aside from the increased antioxidant capacity, was the preservation of the red blood cell (RBC) count, which was significantly decreased in the control group. Stabilizing effects of vitamin C on packed whole blood was recently described [32], as well as beneficial effects on the rheology of RBCs, which were able to pass constricted blood vessels during impaired microcirculation when treated with vitamin C [33]. We assume that the effect of vitamin C on the RBCs is one of the main effects which might contribute to improved kidney function during in vitro kidney perfusion, especially as a counterpart to the impaired microcirculation in I/R-settings, but the exact mechanisms have to be further elucidated. A possible negative effect of vitamin C is hemolysis, which was shown in glucose-6-phosphate dehydrogenase (G6DP)-deficient patients [34], however, we did not detect increased hemolysis in comparison to the control group.

It remains to be determined whether the observed benefits of vitamin C treatment of kidney grafts are enhanced if application of vitamin C is started before the ischemic period. However, while there is no legal limitation to add medication to normothermic machine perfusion systems or apply vitamin C to organ recipients, there might be restrictions for the medication of donors prior to organ retrieval.

Our study had several limitations, the first being the small number of animals and kidneys. Nevertheless, the sample size was sufficient to observe statistically significant effects in some minor parameters. The extrapolation of the results to clinical relevance remains debatable, although we could show effects which were also described in clinical applications of vitamin C, such as hypernatremia. A real baseline value for the ORP and AC measured in the donor animals, prior to kidney explanation is missing in our experiments and will be included in following studies. Furthermore, we used UV-protected syringes and infusion lines for the application of vitamin C to the extracorporeal circuit, however the system itself consisted of non-UV-protective material.

In clinical practice, it is still debated by experts, whether vitamin C treatment is beneficial for kidney function. In a meta-analysis including 1.536 patients performed in 2013 by Sadat et al., vitamin C decreased the risk for acute kidney injury by 33% (risk ratio 0.672, confidence interval 0.466–0.969, *p* = 0.034) [35]. In contrast, excessive and long-term vitamin C consumption might lead to oxalate nephropathy, as described in several case reports [22,24,36,37].

A high-dose intravenous vitamin C regimen of 66 mg/kg/hour for 24 h reduced fluid requirements, improved oxygenation and shortened duration of mechanical ventilation in 37 severe thermally injured patients [25]. In a retrospective analysis, the same intervention led to a reduction of resuscitation fluid volume, increased urine output and trends towards decreased and shortened overall vasopressor requirement [38] However, this therapy was associated higher urine output per day and per hour, but also with an increased risk of renal failure in a retrospective case-control study [39]. No influence of vitamin C on kidney function was observed in two small clinical pilot trials [40,41].

Additionally, it is still debated, which concentration of vitamin C is beneficial, as in theory, it can act as pro-oxidant in presence of redox-active metal ions, which was postulated to lead to the formation of hydroxyl radicals and thus negate any beneficial effects. However, this phenomenon is unlikely to occur in human biology under physiological circumstances [42,43,44].

## 5. Conclusions

Vitamin C treatment drastically reduced oxidative stress of normothermic perfused porcine kidney grafts and preserved red blood cells as oxygen carrier in the perfusate. Thus, adding vitamin C to clinical protocols for kidney graft perfusion might possibly be a beneficial strategy to reduce ischemia and reperfusion related donor organ injury. In this small, preclinical study, the clinically relevant parameters were not improved by the administration of vitamin C. Nevertheless, our results encourage further research to determine if the change in laboratory values detected in this experimental setting will translate into clinical effects. The ideal dosing of antioxidants adapted to the individual patients needs further investigation and, if beneficial, could contribute to improvement of organ preservation techniques.

## Abbreviations

**AC**	Antioxidant Capacity
**BW**	Body Weight
**HB**	Hemoglobin
**IL**	Interleukin
**I/R**	Ischemia/Reperfusion
**NMP**	Normothermic Machine Perfusion
**ORP**	Oxidation reduction potential
**RBC**	Red blood Count
**TNF**	Tumor necrosis factor
